# The Role of Microorganisms in the Development of Breast Implant-Associated Anaplastic Large Cell Lymphoma

**DOI:** 10.3390/pathogens12020313

**Published:** 2023-02-14

**Authors:** Mario Alessandri-Bonetti, Tiffany Jeong, Luca Vaienti, Carolyn De La Cruz, Michael L. Gimbel, Vu T. Nguyen, Francesco M. Egro

**Affiliations:** 1 Department of Plastic Surgery, University of Pittsburgh Medical Center, 1350 Locust St, Suite G103, Pittsburgh, PA 15219, USA; 2 Department of Reconstructive and Aesthetic Plastic Surgery, University of Milan, I.R.C.C.S. Istituto Ortopedico Galeazzi, 20161 Milan, Italy

**Keywords:** breast lymphoma, anaplastic large cell lymphoma, breast reconstruction, breast augmentation, textured implant, breast implant, biofilm

## Abstract

Breast implant-associated anaplastic large cell lymphoma (BIA-ALCL) is a variant of anaplastic large cell lymphoma (ALCL) associated with textured-surface silicone breast implants. Since first being described in 1997, over 1100 cases have been currently reported worldwide. A causal relationship between BIA-ALCL and textured implants has been established in epidemiological studies, but a multifactorial process is likely to be involved in the pathogenesis of BIA-ALCL. However, pathophysiologic mechanisms remain unclear. One of the hypotheses that could explain the link between textured implants and BIA-ALCL consists in the greater tendency of bacterial biofilm in colonizing the surface of textured implants compared to smooth implants, and the resulting chronic inflammation which, in predisposed individuals, may lead to tumorigenesis. This review summarizes the existing evidence on the role of micro-organisms and rough surface implants in the development of BIA-ALCL. It also provides insights into the most updated clinical practice knowledge about BIA-ALCL, from clinical presentation and investigation to treatment and outcomes.

## 1. Introduction

Breast implant-associated anaplastic large cell lymphoma (BIA-ALCL) is a variant of anaplastic large cell lymphoma (ALCL) associated with textured-surface silicone breast implants [1,2,3,4]. It is now recognized as an anaplastic kinase-negative (ALK-), CD30 + lymphoma, a distinct T-cell–derived lymphoma within the non-Hodgkin’s lymphoma spectrum [5]. This neoplasm may present with different clinical phenotypes. A small subgroup of cases presents with solid tumor mass and progressive disease, while most present with delayed-onset (>1 year from surgery) periprosthetic seroma and follow an indolent course, often with a good prognosis [6,7].

BIA-ALCL was first described in 1997 by Keech and Creech, who took part in the treatment of a 41-year-old female presenting with a small mass in the lateral aspect of the right breast five years after breast implant placement [8]. Since first being described, over 1100 cases have been reported worldwide [9]. Mean time from breast implant placement to diagnosis is around 10 years [10].

Although originally believed to be of very rare occurrence, recent studies suggest that BIA-ALCL incidence could be higher compared to previous epidemiological estimates. Current reported incidence ranges between one 1 in 355 and 1 in 30,000 people with a textured surface breast implant and seems to vary further according to manufacturer-specific risks [10,11,12,13].

The disease remains equally distributed among cosmetic and reconstructive patients, suggesting that a history of previous malignancy (e.g., breast cancer) cannot be considered as a risk factor for the development of BIA-ALCL [10,11].

In the past years, the growing scientific evidence on the topic led to the U.S. Food and Drug Administration releasing warning statements and updates associating breast implants with BIA-ALCL [9,14]. As a consequence, media coverage on breast implants and BIA-ALCL is significantly impacting public perception and awareness, causing confusion between facts and suppositions, but also increasing the need for further understanding of this matter [15].

After more than two decades of research, the molecular mechanisms responsible for the aberrant T-cell clonal expansion in BIA-ALCL remain poorly understood [1]. However, to date, consensus on the implant texturization as a known modifiable risk factor and its implication in the development of BIA-ALCL has been reached [16]. Nevertheless, we are still facing a knowledge gap in pathophysiologic mechanisms for developing BIA-ALCL [16].

Several hypotheses have been proposed regarding the etio-pathologic pathways leading to BIA-ALCL, possibly working in concert, including implant immunogenicity due to silicone or its degradation products from textured implants, a genetic susceptibility altering the host autoimmune response, an IL-13 associated allergic response, or chronic inflammation triggered by bacterial biofilm [17,18,19,20].

This article reviews the existing evidence on the role of micro-organisms in the development of BIA-ALCL.

## 2. Textured Surface Implants and BIA-ALCL

Textured implants were introduced in 1968 [21]. Modern textured surface implants date back to the 1990s [22]. Their worldwide success and distribution were justified by their potential role in minimizing capsular contracture risk and reducing implant malrotation, two of the most common and troublesome complications of breast implants [22,23].

Ultimately, textured implants were not found to sufficiently reduce capsular contracture, a process in which the mechanisms remain unclear [24,25]. Nevertheless, bacterial biofilm is likely to play a role in the pathogenesis of capsular contracture [26,27,28,29]. An analysis of removed capsules demonstrated that 17 out of 19 cultures obtained from patients with significant contracture yielded positive results for bacterial growth (mainly coagulase-negative staphylococci), compared with only one out of eight samples obtained from patients with minimal or no contracture [28]. In addition, 14 of the 17 positive cultures from significantly contracted breasts yielded coagulase-negative staphylococci. Subclinical infection or bacterial biofilm may trigger the immune reaction to the secretion of profibrotic cytokines and subsequent contracture [28]. In addition, further evidence demonstrated the benefits of intraoperative antiseptic irrigation of the implant leading to a reduction of the incidence of capsular contracture [30,31].

A causal relationship between BIA-ALCL and textured implants has been established due to the consistent findings in epidemiological studies [16,32]. To date, there has not been any definitively confirmed BIA-ALCL in patients who underwent placement of smooth-implant only [6,33]. Although several reports of patients with smooth implants at the time of BIA-ALCL diagnosis have been published, they all had either a history of textured implant/expander or incompletely known history of previous breast implants [16]. Macro-texturization has also been associated with higher risk of BIA-ALCL when compared to lower grade of texturization [34,35,36].

The type of texturization varies between single manufacturers’ processing of the outer shell of the implant [34]. Among textured devices, Biocell (Allergan Aesthetics, An Abbvie Corporation, Irvine, CA, USA) and Silimed polyurethane (Silimed Corporation, Rio De Janeiro, Brazil) implants showed higher odds ratio of BIA-ALCL incidence compared to Siltex (Mentor, A Johnson & Johnson Company, New Brunswick, NJ, USA). According to Valencia-Lazcano et al. [37], implant-specific risk of BIA-ALCL was 1:2832 for Silimed polyurethane, 1:3345 for Biocell and 1:86,029 for Siltex implants. One prospective study at a single institution reported even higher risk for Allergan Biocell implants, with 1:355 women developing BIA-ALCL [11]. A time-to-event analysis indicates that the risk of developing BIA-ALCL increases over time [38]. However, existing studies have not assessed the prophylactic value of textured device explantation, with or without capsulectomy, in preventing this disease process. Moreover, current FDA guidelines do not recommend replacing or removing textured implants in asymptomatic patients [39].

Despite the effort of some authors to classify breast implants based on type of implant texturization, all focus only on the device physical properties without addressing biological properties and none of the classification attempts has been clinically validated and universally accepted [16]. The International Organization for Standardization (ISO) updated in 2018 its breast implant classification, which currently is the most used classification system, only accounting for average surface area and roughness through scanning electron microscopy, a relatively nonspecific characterization of texturization [16,34]. Thus, there is room for improvement for a clinically validated classification system that includes parameters beyond “surface roughness” [32,33,34]. Indeed, some authors claim that the incidence per manufacturer does not always correlate with surface roughness and area and therefore the different manufacturing process should also be considered [36]. Increasing attention is being paid to the host’s reaction to the implanted device [32]. In animal models, different implant surfaces were associated with variable host inflammatory response (e.g., less rough surfaces induced weaker inflammatory response than rougher) [40], suggesting the need to quantitatively and qualitatively standardize breast implants according to the immune reaction in humans.

## 3. Role of Bacterial Biofilm and Chronic Inflammation in BIA-ALCL

One of the hypotheses that could explain the link between textured implants and BIA-ALCL consists in the greater tendency of bacterial biofilm in colonizing the surface of textured implants compared to smooth implants and the resulting chronic inflammation which, in predisposed individuals, may lead to tumorigenesis [2,3,5,27].

Biofilms are surface-associated microbial colonies, embedded in a self-secreted extracellular matrix [41]. A significant property of biofilm is the increased resistance to phagocytosis and antiseptics, which, in a clinical context, implies resistance to host defense mechanisms and antimicrobials. Consequently, bacterial biofilm is able to chronically stimulate the host immune system and its inflammatory process [26,41,42].

Several in vitro studies have reported that rough-textured breast implants promoted the increase of propensity for biofilm growth compared to smooth surface implants [43,44,45,46]. This difference is felt to be related to the larger surface area and increased bacterial adhesion (*Staphylococcus epidermidis*) attaching to rough surfaces [43]. James et al. confirmed that rougher breast implants with more surface (i.e., Siltex and Biocell) harbored more bacterial biofilm (*Staphylococcus epidermidis, Pseudomonas aeruginosa* and *Ralstonia pickettii*) than smoother implants [45]. Jones et al. demonstrated a prominent positive correlation between implant surface area and bacterial attachment/growth biofilm (*Staphylococcus epidermidis, Staphylococcus aureus,* and *Ralstonia pickettii*) [46]. Similar results in biofilm (*Staphylococcus epidermidis, Staphylococcus aureus*) and breast devices were obtained by Lee et al. [44].

Bacterial biofilm likely plays a role in the pathogenesis of capsular contracture [25,26,29]. Regardless of the source, either endogenous or introduced during surgery [47], bacterial biofilm creates a chronic inflammatory environment and induces the recruitment of macrophages and myofibroblasts which contribute to fibrous capsule formation around the implant [28,48]. Although no correlation between capsular contracture and BIA-ALCL has been verified, the chronic stimulation of the immune response by peri-prosthetic bacterial colonization, as likely seen in capsular contracture, leads to the association of bacterial biofilm and BIA-ALCL.

Other examples of chronic inflammation-driven lymphomas have been described in the literature. There is evidence that cutaneous T-cell lymphomas are preceded by chronic inflammation [49]. Similarly, the biological peptide gluten, in combination with the intestinal microbiome, has been shown to drive T-cell changes in patients with celiac disease towards T-cell lymphoma transformation [50]. Lastly, the gastric mucosa-associated B-cell lymphoma that arises from a chronic inflammatory reaction to Helicobacter pylori is another example [51].

In support of the chronic inflammation hypothesis, Lechner et al. [52] detected high production of T- cell-associated cytokines IL-6 and IL-10 in their BIA-ALCL model. Kadin et al. [19] confirmed a Th17/Th1 phenotype of BIA-ALCL neoplastic lymphocytes, supporting the potential role of antigenic stimulation and chronic inflammation. Wolfram et al. [53] showed intracapsular T cells producing IL-17, IFN-Υ, IL-6, IL-8 and TGF-β, confirming a Th17/Th1 weighted local immune response in silicone implants with capsular fibrosis. Hu et al. [54] showed a linear correlation between implant capsular bacterial load and the proliferation of activated lymphocytes. The correlation was strongest for CD4+ T cells, the same phenotype found in BIA-ALCL aberrant cells [54]. In a subsequent study evaluating the microbiome of BIA-ALCL and non-tumor capsule samples, Hu et al. [20] reported a gram-negative shift in BIA-ALCL samples, with significantly greater proportions of gram-negative bacilli *Ralstonia* spp. On the contrary, *Staphylococcus* spp., a normal constituent of skin and endogenous breast microflora, was the most frequently identified organism on the contralateral benign breast capsules. Gram-negative bacteria have a lipopolysaccharide coat (LPS) which is a known powerful trigger of the host immune system. Reinforcing the findings of Hu et al., Mempin et al. [55] showed a unique, proliferative response of patient-derived BIA-ALCL primary tumor cells to the presence of Gram-negative bacterial LPS. This behaviour was not evident for tumor cells obtained from phenotypically similar cutaneous form of ALCL, a T-cell leukemia cell line (MT-4), and peripheral blood mononuclear cells derived from patients who have been diagnosed with capsular contracture and from those who have not been previously exposed to breast implants [55]. Moreover, the proliferative response was absent in the peripheral blood mononuclear cells from BIA-ALCL patients, suggesting the response to LPS is a local tumor response and not a general systemic response [55].

However, Walker et al. [17] used 16S rRNA sequencing to test the *Ralstonia* hypothesis and investigated the microbiome of BIA-ALCL specimens and benign breast implant capsules from the contralateral breast. Their study failed to replicate the *Ralstonia* species data previously described by Hu and colleagues [20]. Walker et al. [17] demonstrated a Gram-positive predominance and that BIA-ALCL does not appear to have a distinct microbiome in comparison to normal capsules, challenging the notion of a Gram-negative shift in BIA-ALCL capsules.

Furthermore, it should be recognized that Ralstonia species are well known contaminants that may confound results of assays surveying PCR-amplified marker genes of microbial communities [56,57]. These species are ubiquitous water and soil organisms that have been found in molecular biology grade water, PCR reagents and even in DNA extraction kits [56,57,58]. Therefore, failure to replicate studies associating Ralstonia species with BIA-ALCL specimens may highlight the pitfalls of employing these exquisitely sensitive investigative techniques, especially in low microbial mass specimens.

A summary of the available findings on the role of micro-organisms on the pathogenesis of BIA-ALCL is described in Table 1. Of note, findings such as the non-proliferative response of BIA-ALCL cells to breast implant silicone, type of interleukins secreted by intracapsular T cells, and Th1/Th17 weighted inflammatory response are evidence of an inflammatory response to an antigen. Although this may be the result of infection pathogens, the mechanistic evidence connecting pathogens to inflammation is still missing.

**Figure 1:**
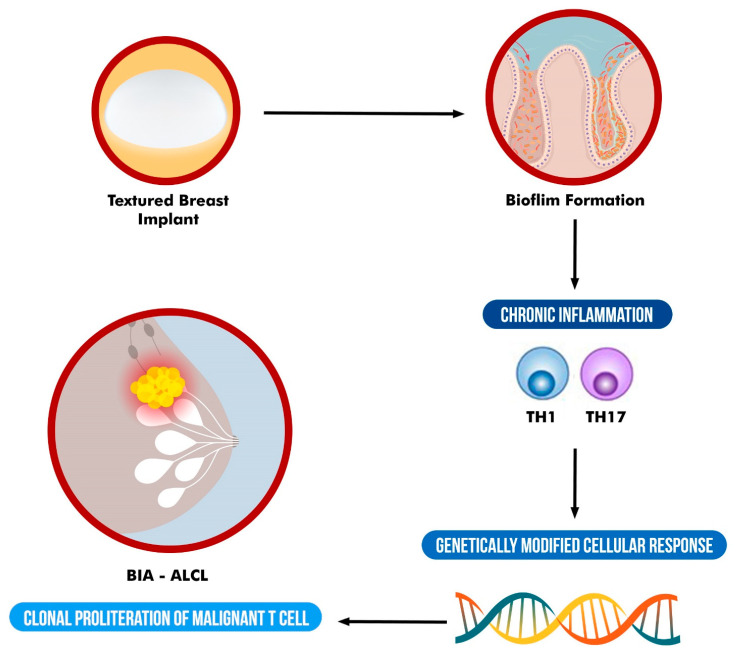
Schematic representation of the suggested multifactorial etiology of BIA-ALCL.

Although evidence for a specific bacterial pathogen remains elusive, that does not preclude involvement of an infectious agent. In addition, although some data support a bacterial role in the pathogenesis of BIA-ALCL, it has not achieved universal acceptance and rather should be considered as a multifactorial process which involves other factors such as genetics (Figure 1). Further research on the origin of BIA-ALCL is needed.

## 4. Genetics and BIA-ALCL

It is very likely that chronic inflammation plays a prominent role in the etiological pathways of BIA-ALCL development. However, a genetic susceptibility is also believed to play a key role in the process of BIA-ALCL tumorigenesis. The presence of bacteria, coupled with a unique genetic background of the host, such as HLA variations [59], could explain the relatively uncommon incidence of BIA-ALCL, as it requires both bacterial presence and genetic susceptibility to cause ongoing immune activation and malignant transformation in susceptible hosts over time.

A study from de Boer et al. showed that BRCA 1/2 mutation carriers with implants have an increased risk of BIA-ALCL [13]. Moreover, Ionescu et al. found that the absolute risk of developing BIA-ALCL in women with BRCA 1/2 mutations with breast implants was 1/1551 at 75 years of age compared with 1/7507 in women from the general population [60]. Women diagnosed with Li-Fraumeni syndrome, a cancer-predisposing condition caused by germline pathogenic mutations in *TP53*, have been reported to develop BIA-ALCL, perhaps suggesting that they may also be at greater risk [61,62].

The JAK-STAT pathway has been shown to mediate inflammation-associated cancers and been postulated to have a key role in BIA-ALCL [63]. The JAK-STAT pathway regulates embryonic development and signaling and is implicated in cell proliferation, differentiation and apoptosis [13,64]. The pathway is dysregulated across various types of T-cell lymphomas; however, the extent of deregulation is significantly higher in BIA-ALCL [13,65,66] and it was present in 60% of cases according to a recent study [63]. To date, oncogenic JAK- STAT3 pathway mutations have been described in 43.8 percent of successfully tested cases [1]. Blombery et al. also performed targeted next-generation sequencing on 11 BIA-ALCL specimens and found that 10 of the 11 cases were associated with a JAK-STAT3 pathway genetic variant. Additionally, 7 of the 11 cases contained a *STAT3* variant [67].

## 5. Implications for Clinical Practice

### 5.1. Clinical Presentation

The most common BIA-ALCL presentation consists of painless swelling of the breast and late-onset seroma. A minority of patients presents with a palpable mass in the breast [68,69]. Other signs and symptoms may be present, such as capsular contracture, local pain, lymphadenopathy and B-symptoms [6,68,69]. Patients who had implants placed for cosmetic augmentation and those who underwent implant-based reconstruction showed no difference in both incidence and clinical presentation [33]. A systemic review by Sharma and et al. reported that 82% of patients presented with swelling in the breast and breast asymmetry, 10% with pain and 8% with palpable breast mass [69]. Less frequent presentations of BIA-ALCL include regional lymphadenopathy, local skin rash, fever and capsular contracture [7,70]. Other possible causes of seroma, such as trauma or infection, should be explored alongside BIA-ALCL diagnosis [6,7].

### 5.2. Investigation

According to the 2022 National Comprehensive Cancer Network guidelines, any textured implant carrying-patient who presents with a delayed-onset (>1 year after implantation) seroma, or with a periprosthetic solid mass, deserves prompt investigation for BIA-ALCL [71] Breast ultrasound, MRI or PET-CT in selected cases represent initial workup [71]. Early diagnosis is best achieved by cytological analysis of the first seroma aspirate (minimum 50 mL), as repeat seroma aspirates may dilute the neoplastic cells with new seroma fluid [70,71,72]. Cytological analysis should occur in conjunction with breast imaging and should include CD30 immunohistochemistry and T Cell quantification and characterization. A mass may suggest a more severe presentation, warranting needle or open biopsy [7,70,72].

BIA-ALCL’s clinical behavior resembles more solid tumors than other non-Hodgkin lymphomas. Therefore, the MD Anderson Cancer Center TNM developed a more appropriate staging system which replaced the Ann Arbor classification for non-Hodgkin lymphomas [69,72].

### 5.3. Treatment

Although most of the data on treatment and outcomes come from retrospective case series and reports, factors associated with higher risk of recurrence and mortality include presentation with a mass, advanced stage at presentation, extracapsular involvement and incomplete surgery [33,68,69]. Complete surgery is defined as breast implant removal and total capsulectomy with complete excision of any associated mass and negative margins on final pathologic evaluation [6]. In some cases, mastectomy has been performed at the time of capsulectomy [69]. However, there is no data supporting an improvement in BIA-ALCL clinical outcomes with mastectomy. Patients with stage 1 BIA-ALCL and tumors completely confined to the capsule may pursue immediate breast reconstruction, with most patients achieving complete remission and high patient satisfaction [73]. Patients with more advanced BIA-ALCL presentation may be considered for reconstruction following a 6–12-month delay [73]. In 2022, the National Cancer Center Network provided guidelines to diagnosis and treatment of BIA-ALCL [71]. In localized BIA-ALCL, complete surgical excision is the landmark treatment, demonstrating improvement in event-free survival and overall survival compared to limited surgery, chemotherapy, or radiation therapy [6]. Systemic therapy is appropriate for more advanced cases or incomplete surgery [71]. Although large trials evaluating the effectiveness of different chemotherapy protocols in BIA-ALCL are lacking, the NCCN suggests the use of Bremtuximab vedotin, CHOP (Cyclophosphamide, Hydroxydaunorubicin, Oncovin, Prednisone) or their combination as systemic treatment regimens [71], similarly to primary cutaneous or systemic ALCL [74]. Radiation therapy could also be considered in case of incomplete capsulectomy or nodal involvement [71]. A systematic review revealed that 39% of all BIA-ALCL patients received adjuvant chemotherapy and 37% received adjuvant radiotherapy [69]. Of note, 100% of patients with stage 4 disease received chemotherapy and radiotherapy, while patients with stage 1 received chemotherapy (23%) and radiotherapy (27%) in fewer cases [69]. This highlights the importance of accurate staging using TNM, which has been predicative of prognosis in BIA-ALCL [6,70]. NCCN guidelines also underscore that because BIA-ALCL has been found incidentally in the contralateral breast in 4.6% of cases surgeons should consider removal of the contralateral implant prophylactically [6,70].

### 5.4. Outcomes in BIA-ALCL

Although most patients with BIA-ALCL have a relatively indolent clinical course, reports of deaths attributable to the disease emphasize the importance of a timely diagnosis and adequate treatment with appropriate surveillance [6]. Indeed, Clemens et al. demonstrated that the overall survival for BIA-ALCL was 94% at three years and 91% at five years. Earlier stage at presentation is associated with higher survival rates [6,69]. Therefore, early diagnosis and treatment are critical in optimizing outcomes [33]. Complete capsulectomy with clear margins has been demonstrated to be the most effective treatment in improving clinical outcomes [6,69]. Recently, Tevis et al. [68] prospectively reported outcomes in a cohort of 52 women with cytologically proven BIA-ALCL over a five-year period. Among the study population, two patients presented with distant metastases and 14 patients presented with nodal involvement. Most patients presented with stage 1 or 2 disease. Although two patients (3.8%) experienced tumor recurrence, eventually all patients achieved complete remission [68]. The 2022 NCCN recommends surveillance following response to treatment every 3–6 months for 2 years, and then as clinically indicated [71]. The NCCN also endorses imaging surveillance no more often than every 6 months for 2 y and then annually for 5 years or as clinically indicated [71].

### 5.5. The Role of Antiseptic Solutions

The pathogenetic involvement of subclinical infection and chronic inflammation in BIA-ALCL origin warrants evidence-based guidelines for intraoperative strategies to mitigate biofilm formation in attempt to reduce the risk of BIA-ALCL [75]. One potential preventive strategy is the use of antiseptic solutions for pocket irrigation. However, studies have demonstrated a lack of consensus on best practices for implant and pocket irrigation solutions. In an American Society of Plastic Surgeons survey, triple antibiotic solution (TAB; bacitracin, cefazolin, and gentamicin) without Betadine was used by 41% of plastic surgeons, while the remaining 59% favored 29 other irrigation solution regiments [76]. However, preliminary in vitro evidence suggests that TAB may be ineffective in killing bacterial species seen in breast implant biofilm [76,77,78]. Many studies endorse the use of povidone-iodine pocket irrigation for its widespread antimicrobial activity, low cost and efficacy in dissolving biofilms [30,78,79,80,81]. Furthermore, Betadine may be preferable to other antibiotic irrigation solutions because of its low immunogenicity and no documented evidence of bacterial resistance [78,82]. There is no standardized formulation of Betadine used as an irrigation solution. However, 25–50% Betadine has been demonstrated to balance concerns over high concentration and achieving the best broad-spectrum antimicrobial impact [78,83]. While evidence on the potential association between any specific micro-organism and BIA-ALCL implants remains inconclusive and hypothetical, Betadine does confer greater gram-negative coverage than TAB and has been posited as the preferred antimicrobial [82,83]. In vitro results suggest that Betadine may be used in conjunction with TAB to enhance TAB efficacy if the pocket is treated for at least 5 min [78,79]. Other studies propose that hypochlorous acid or derivatives are efficacious in antimicrobial activity during antiseptic breast irrigation [77,84]. While this antiseptic is widely used in other surgical procedures, data is limited on the efficacy of hypochlorous acid in breast reconstruction. It is important to note that no studies have demonstrated the effects of disinfection protocols on risk of BIA-ALCL, and most of the available literature examining pocket irrigation efficacy focus on capsular contracture as the primary outcome [85]. Further studies linking implant and pocket irrigation techniques to BIA-ALCL incidence are warranted.

## 6. Conclusions

Current evidence suggests a multifactorial etiology of BIA-ALCL, namely the combination of bacterial colonization, chronic inflammation, and genetic susceptibility. Current evidence suggests that textured implants are heavily implicated in the BIA-ALCL pathogenesis. Therefore, it is essential to weigh risks and benefits of this implant choice and to allow patients to make an informed decision prior to surgery. Many surgeons in the United States have moved away all together from textured implants because of the increased risk associated. However, given the incomplete and fragmentary understanding of BIA-ALCL pathogenesis and given the paucity of available studies, further research is needed. The scientific community would benefit from greater scrutiny of the infectious presence within the breast capsule of BIA-ALCL specimens to identify the incidence of specific pathogens. Further studies would be needed to assess the impact of antiseptic and antimicrobial agents in minimizing the incidence of BIA-ALCL by reducing the contamination of the implant. Mechanistic studies assessing the link between the potential antigen, specific genetic sequences and development of BIA-ALCL will be useful to extend our knowledge on the topic. Surely, there is a need to further elucidate mechanisms at the base of this rare, yet serious condition.

Although much still has to be done, the international community has united to establish evidence-based guidelines in an attempt to offer guidance on the optimal management of BIA-ALCL in a joint effort to improve our understanding and patient outcomes.

## Figures and Tables

**Table 1:** Summary of the available findings compatible with and against the hypothesis of a microbial involvement in the pathogenesis of BIA-ALCL.

	PROS	CONS
Micro-organisms	Gram-negative bacilli *Ralstonia* spp shift in BIA-ALCL samples [20]. Gram-positive predominance in BIA-ALCL specimens [17].	Failure to replicate the *Ralstonia* hypothesis using 16S rRNA sequencing [17]. *Ralstonia* species have been found in contaminants [56]. BIA-ALCL does not appear to have a distinct microbiome in comparison to normal capsules [17].
Lipopolysaccharide (LPS)	Proliferative response of patient-derived BIA-ALCL primary tumor cells to the presence of Gram-negative bacterial LPS. This behavior was not seen in other types of ALCL [55].	
Silicone	Breast implant shell of all surface grades alone does not produce a proliferative response of BIA-ALCL cells, indicating that breast implant alone does not act as a pro-inflammatory stimulant [55].	
Lymphocytes	Linear correlation between implant capsular bacterial load and the proliferation of activated lymphocytes. The correlation was strongest for CD4+ T cells, the same phenotype found in BIA-ALCL aberrant cells. [54] Th17/Th1 phenotype of BIA-ALCL neoplastic lymphocytes [19].	
Interleukins	Intracapsular T cells producing IL-17, IFN-Υ, IL-6, IL-8 and TGF-β, confirming a Th17/Th1 weighted local immune response [53].	

## Data Availability

Not applicable.
